# Bacterial Flagellar Filament: A Supramolecular Multifunctional Nanostructure

**DOI:** 10.3390/ijms22147521

**Published:** 2021-07-14

**Authors:** Marko Nedeljković, Diego Emiliano Sastre, Eric John Sundberg

**Affiliations:** Department of Biochemistry, Emory University School of Medicine, Atlanta, GA 30322, USA; marko.nedeljkovic@emory.edu (M.N.); dsastre@emory.edu (D.E.S.)

**Keywords:** bacterial flagella, flagellin, filament, FliD

## Abstract

The bacterial flagellum is a complex and dynamic nanomachine that propels bacteria through liquids. It consists of a basal body, a hook, and a long filament. The flagellar filament is composed of thousands of copies of the protein flagellin (FliC) arranged helically and ending with a filament cap composed of an oligomer of the protein FliD. The overall structure of the filament core is preserved across bacterial species, while the outer domains exhibit high variability, and in some cases are even completely absent. Flagellar assembly is a complex and energetically costly process triggered by environmental stimuli and, accordingly, highly regulated on transcriptional, translational and post-translational levels. Apart from its role in locomotion, the filament is critically important in several other aspects of bacterial survival, reproduction and pathogenicity, such as adhesion to surfaces, secretion of virulence factors and formation of biofilms. Additionally, due to its ability to provoke potent immune responses, flagellins have a role as adjuvants in vaccine development. In this review, we summarize the latest knowledge on the structure of flagellins, capping proteins and filaments, as well as their regulation and role during the colonization and infection of the host.

## 1. Introduction

The bacterial flagellum is one of the most complex and dynamic biological nanomachines known and has attracted attention since its discovery in the late nineteenth century. The history of flagellar research is an excellent example of the gradual transition of biological studies, that were purely morphological, to biochemical and biophysical studies at the atomic level, allowing current understanding to the point of being able to modify it for various purposes.

Bacterial flagella are appendages on the cell body that provide motility. The number of flagella per cell varies depending on the species. Monotrichous species have only one flagellum at the pole (e.g., *Pseudomonas aeruginosa*); lophotrichous have multiple flagella at the same pole (e.g., *Helicobacter pylori*); amphitrichous have one flagella at each pole (e.g., *Campylobacter jejuni*); and peritrichous bacteria have multiple flagella in all directions (e.g., *Escherichia coli, Salmonella enterica*) [1]. Flagella consist of three morphologically distinct sections: a membrane-embedded basal body, a hook and a filament [2]. The basal body is a complex assembly that functions as a motor powered by a proton gradient; its rotation generates torque. This torque is transmitted through the hook to the filament that serves as a propeller, creating thrust and pushing bacteria through liquid environments [3]. Counterclockwise rotation of the motor provides smooth forward swimming, and in peritrichous bacteria filaments form a bundle on one pole of the cell [4]. Clockwise rotation of some of the flagella results in unraveling of the bundle and specific tumbling movement. Filaments are much longer than the cell body and during smooth swimming they adopt a left-handed supercoiled corkscrew shape, while clockwise rotation changes the handedness [5,6].

In species such as *Vibrio cholerae* and *H. pylori*, flagella are enclosed by a sheath—a membranous structure contiguous with the outer membrane with various proposed functions, such as the prevention of filament disintegration in gastric acid, immune evasion and the place of origin of the outer-membrane vesicles that protect bacteria from bacteriophages [7]. Spirochetes are a special case since they have periplasmic flagella, the rotation of which causes undulation of the cells [8].

The most extensively studied flagellar systems by far are those of *E. coli* and *S. enterica* ser. Typhimurium and, with some exceptions, they serve as models to explain flagellar architecture in general. During the assembly of the flagellum, integral membrane proteins of the basal body use the Sec pathway for insertion into the membrane. Structural components of the periplasmic rod, the hook and the filament are transported by a type 3 secretion system (T3SS) specifically adapted to carry the distal flagellar components across the membrane. The flagellar T3SS is located at the base of the flagellum and it is one of the earliest structures of the assembly, together with the MS ring of the basal body. The structure and mechanism behind basal body function were a subject of several recent reviews [9,10,11,12], and they will not be considered further here. Instead, we will focus on the various aspects of the flagellar filament, its components and the role it plays in host–pathogen interactions.

## 2. Historical Overview

During his work, which confirmed the germ theory, Robert Koch also made the first micrographs of the bacterial flagella [13]. By the 1930s there was a consensus among bacteriologists that these were locomotory organelles and a brief controversy caused by the South African bacteriologist Adrianus Pijper in the mid-1940s sparked a series of biochemical and electron microscopy studies that finally settled the question of the role of bacterial flagella [14].

The first suggestion that flagella are of a proteinaceous nature came from Boivin and Mesrobeanu in 1938, based on the observed insolubility of the H antigen (a historical designation of the flagellar antigen) in trichloroacetic acid, together with its thermolability and sensitivity to alcohol [15]. The majority of the early biochemical studies of flagella were conducted by Claes Weibull, a student of Arne Tiselius at the University of Uppsala at the time. Using a purification protocol developed by his colleague Sven Gard that he further improved, he mainly studied flagellar filaments of *Proteus vulgaris* and *Bacillus subtilis*, combining various approaches such as paper chromatography, electrophoresis, ultracentrifugation and light absorption [16,17,18,19]. His analyses showed that flagella are protein structures that could be decomposed by acid treatment into smaller particles of elongated shape and molecular weight of approximately 41 kDa [16]. Henry Koffler and coworkers at Purdue University took an interest in the matter and confirmed those findings [20,21,22]. Weibull and William Astbury performed the first X-ray analysis of flagella at the University of Leeds in 1949 and obtained a diffraction pattern characteristic for fibrous proteins [23]. In 1955, they proposed the name “flagellin” that was adopted for the protein molecules that make up the flagellar filament [24].

In the following years, several groups reported successful attempts to repolymerize flagellin into native-like filaments by varying pH, salt concentration and temperature [25,26,27]. Additionally, Asakura et al. noticed that the filament elongation in vitro occurs only at the distal end, making the filament growth unidirectional [28]. The same principle was also observed in living cells, raising the question of how this process occurs and what is the energy source that drives it [29].

During the 1960s, much attention was directed to the structural organization of the filament. Kerridge et al. observed that the filament is a hollow structure with the flagellin molecules packed in a helical array [30]. During this time, several models of filament organization were proposed based on the interpretation of electron microscopy and X-ray diffraction data with varying numbers of longitudinal rows of flagellin positioned around the central channel [30,31,32]. Analyzing optical diffraction of the electron micrographs of the *S.* Typhimurium filaments, O’Brien and Bennet finally proposed a correct model for *S.* Typhimurium in 1972 with 11 longitudinal rows of flagellin, now referred to as protofilaments, positioned around a central hole [33].

Another distinctive feature of the filament was noticed in 1985. Ikeda et al. were studying intact flagella in *Salmonella* and observed that their ends are tapered and blunt [34]. On many, they noticed a thin protein layer that looked like a cap. Using immunogold labeling, it was shown that the cap structure at the tip of the filament is made of FliD (also called hook-associated protein 2 or HAP2) [35], a protein isolated and identified as a minor component of the hook several years before [36]. These studies led to the establishment of a model in which the FliD complex initially assembles at the tip of the hook before any of the flagellin molecules are secreted, thus preventing the leakage of flagellin and assisting in the initiation of filament formation. Therefore, although initially part of the hook junction region, with the formation and elongation of the filament beneath the FliD cap, the cap becomes localized to the tip of the filament.

## 3. Flagellins

Flagellins are elongated proteins that constitute the flagellar filament and, apart from the FliD protein at the tip, are the only structural component of the filament. Their molecular weight ranges from 26 kDa in *Bacillus cereus* to 115 kDa in *Desulfotalea psychrophila* [37,38]. In *E. coli* and *S.* Typhimurium flagellins they are typically 51 kDa, although this varies among strains; for example the symbiotic strain *E. coli* Nissle 1917 has a flagellin protein of 61 kDa, while in some *Salmonella* mutants functional flagellins of 41 and 42 kDa have been reported [39,40]. Terminal regions of flagellins are highly homologous among species and they build the core of the filament [38]. Conversely, a hypervariable region in the middle of the polypeptide sequence makes outer domains significantly different, and in some bacteria such as *B.* *subtilis* it is completely absent [38,41].

The flagellin genes are known under various names depending on the species, such as *fliC* in *E. coli*, *P. aeruginosa*, and *Salmonella*, *hag* in *B. subtilis*, and *flaA* in *C. jejuni* and *Helicobacter pylori*. The number of flagellin genes also varies. Approximately 45% of bacteria have more than one flagellin-encoding gene in their genomes, the most extreme case being *Magnetococus* with 15 [42]. While the *E. coli* genome harbors only one flagellin gene, *fliC*, *Salmonella* has two, *fliC* and *fljB* [43]. However, only one of them is expressed at any point; the switch between them is called phase variation [44]. These two proteins diverge in the middle region providing distinct antigenicities. The studies on mutants locked in one of the two phases showed the different swimming properties and advantages of the FliC-expressing strains in colonization and infection of the gastrointestinal tract [45,46].

In other species with two flagellins such as *H. pylori*, *Campylobacter* spp. or *Shewanella putrefaciens*, both are present in the filament, the minor flagellin predominantly in the proximal region, while the major flagellin forms the remainder of the filament [47,48,49,50]. Although knocking out the minor flagellin does not usually affect the length of the filament, mutants exhibit changes in motility, especially in more viscous media, suggesting that having two flagellins in the filament provides optimal swimming characteristics in different environments [50,51]. The minor flagellin of *C. jejuni* also plays a role in defense against bacteriophage infection [52].

At the species level, *P. aeruginosa* has two types of flagellin genes, A and B, which differ in molecular weight and recognition by antibodies [53,54]. They are not, however, simultaneously present in the genome, but rather they are strain-specific with strains such as PAK containing type A FliC, while strains like PAO1 have type B FliC. While their N- and C-termini are highly conserved, the central region of the type A FliC is significantly shorter compared to the type B [55].

The number of flagellins in one cell can be as high as six (*Caulobacter crescentus* and *Vibrio vulnificus*) or even seven (*Rhizobium leguminosarum*) [42,56,57]. In *V. vulnificus,* mass spectrometry confirmed the presence of five distinct flagellins in the filament, with three having higher impact on motility, adhesion and cytotoxicity [56]. Some of these are redundant, such as for *C. crescentus* in which five of six flagellins can make filaments alone [42].

### 3.1. Flagellin Structure

**Figure 1:**
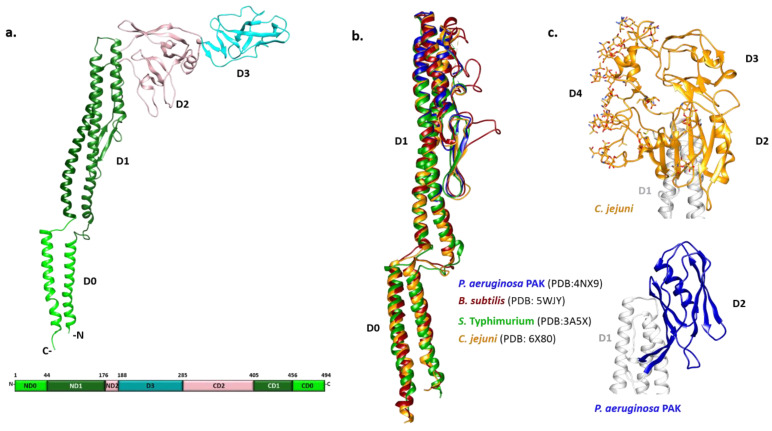
Structural characteristics of flagellins. (**a**) FliC monomer from *S.* Typhimurium with four distinct domain (PDB: 3A5X). Domains D0, D1 and D2 have N- and C-teminal moities, while D3 is formed by a continuous polypeptide sequence in the middle; (**b**) Structural alignment of D0 and D1 domains from various species showing high structural homology among them; (**c**) Outer domains of the *C. jejuni* flagellin FlaA and *P. aeruginosa* PAK type a FliC.

Early biophysical characterization showed that the N- and C-termini of monomeric flagellin are mainly disordered and critical for polymerization, although they become highly structured in the filament [58,59]. Due to the tendency of full-length flagellin to polymerize, crystallization of *S.* Typhimurium flagellin FliC was achieved only after the removal of its terminal regions by limited proteolysis [60]. The overall shape of FliC obtained by combining X-ray crystallography and cryo-EM approaches revealed a structure in the form of a Greek letter Γ with four distinct domains (Figure 1a) [61,62]. Starting from the N-terminus, the polypeptide chain makes a single helix that forms one half of an α-helical domain called D0, continues with the partial formation of domains D1 and D2, and forms domain D3 prior to completing the folds of the previous three domains. Thus, domains D0-D2 have N- and C-terminal moieties.

The N- and C-terminal helices of D0 form a coiled coil in the filament. The organization of D1 with three α-helices and a β-hairpin resembles a four-helix bundle with an extensive hydrophobic core. D2 and D3 consist primarily of β-strands organized in a specific fold called β-foliums in which the tips of the β-hairpins are bent or twisted [61,62].

While all flagellin structures known so far exhibit a high degree of structural similarity in the D0 and D1 region, the structures of the variable regions differ extensively (Figure 1b,c). The structure of the *P. aeruginosa* type A FliC lacking the D0 domain showed that the D1 domain is highly similar to its counterpart in *S.* Typhimurium [63]. Conversely, the D2 domain adopts a different fold with two β-sheets and one α-helix between them forming a less flexible cup-like structure positioned parallel to the D1 domain, instead of pointing away from it as in *Salmonella* (Figure 1c). In the case of *Campylobacter*, the variable region of the flagellin FlaA is larger and forms three domains (D2, D3 and D4) (Figure 1c) [64]. The D2 and D3 domains of the *Campylobacter* FlaA are structural homologs of each other and of the *Pseudomonas* FliC D2. D4 is inserted between C-terminal moieties of D2 and D1, with most of the FlaA glycans located on this domain. It is the most exposed outer domain resembling a shield for both D2 and D3 and, contrary to them, D4 does not have structural homologs.

### 3.2. Posttranslational Modifications of Flagellins

The presence of posttranslational modifications in flagellins was suspected early on, but their extent and importance became apparent only recently. Investigating discrepancies between theoretical and observed molecular weights showed that *Pseudomonas* type A flagellin was glycosylated [55]. A genomic island between *flgL* and *fliC* genes containing 14 open reading frames (ORFs) is responsible for glycosylation and it specifically modifies type A flagellin, while having no effect on type B [65]. These glycan chains in the PAK strain are *O*-linked to residues T189 and S260 through rhamnose and vary in length (up to 11 monosaccharides) and composition [66].

The type B flagellin characteristic of the PAO1 strain also contains *O*-linked glycans at serine residues 191 and 195, although in this case it is a much simpler modification with a single monosaccharide [67]. The genomic island responsible for type B glycosylation is smaller than its type A counterpart and contains four ORFs. *Pseudomonas* type A and B mutants in which glycosylation is absent or incomplete do not affect motility in general, although the lack of glycosylation significantly decreased virulence of both PAK and PAO1 strains in a burned-mouse model of infection [68]. Apart from *O*-linked glycans, *N*-linked glycosylation was also reported in *P. aeruginosa* strain PA14, with three sites on the D0 and D1 domains proposed as the most likely candidate positions [69].

*Campylobacter* flagellin is glycosylated at 19 serine and threonine residues with *O*-linked pseudaminic acid or related derivatives [70,71,72]. Glycosylation in *C. jejuni* is responsible for the 6 kDa difference between predicted and experimentally obtained molecular weight. Approximately 50 genes in a locus that is adjacent to the *flaA* and *flaB* genes encode the *Campylobacter* glycosylation machinery [73]. Glycosylation of flagellin in *Campylobacter* is necessary for filament assembly as mutants that produce unglycosylated flagellin do not have filaments and flagellin accumulates intracellularly [74].

*H. pylori* flagellins are also glycosylated with pseudaminic acid with most glycosylation sites in the central core region of the protein [75]. As in *Campylobacter*, glycosylation plays an important role during assembly of the filament; abolishing glycosylation leads to loss of the filament and motility, even though the levels of flagellin mRNA are unaffected [75].

The flagellin of *Listeria monocytogenes* has *O*-linked *N*-acetylglucosamines at up to six sites located in the central surface exposed region of the protein [66]. The flagellin of *P. syringae* is also glycosylated and these modifications are important for its virulence and swarming [76,77].

Another modification that is present in flagellins is methylation. In *S.* Typhimurium, the presence of methylated lysines was reported as early as 1959 [78]. Lysine methylation is performed by the enzyme FliB. Abolishing methylation does not affect filament assembly or motility [79]. However, it was shown that it promotes adhesion and host cell invasion [80]. *N*-methylation was also detected in *Shewanella oneidensis* [81].

## 4. Filament

**Figure 2:**
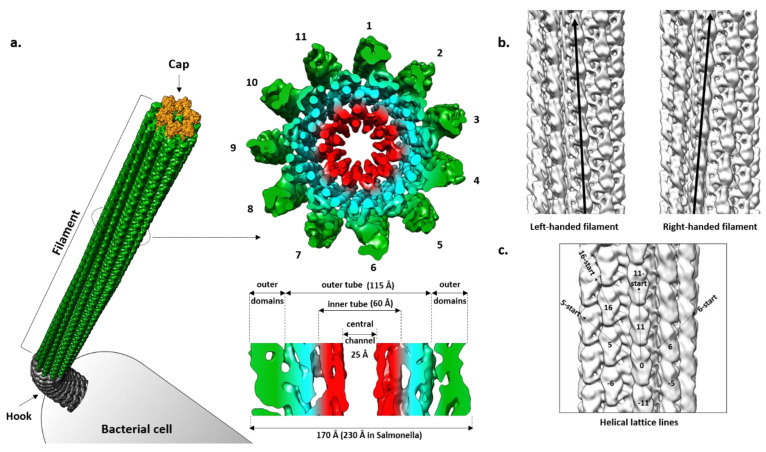
Cryo-EM structure of the flagellar filament from *P. aeruginosa* PAO1. (**a**) Cross-section of the left-handed filament consisting of 11 protofilaments. The inner tube (red) made of D0 domains and outer tube (cyan) together form the densely packed inner core of the filament. Outer domains are shown in green; (**b**) Left- and righ-handed filaments with all protofilaments tilted to the left and right from the central axis; (**c**) Helical symmetry lines in the filament. EMD: 8855, 8856.

The filament is a tubular structure made of more than 20,000 copies of the flagellin protein arranged in a helical fashion [82]. Flagellin is rotated and translated 11 times along the screw axis, making two turns, before it reaches its original position. Such an arrangement results in 11 stacks of flagellin along the axis called protofilaments, which are the basic functional units of the filament (Figure 2a). Depending on the interaction between flagellin molecules in a protofilament, it switches between two different states, left-handed (L) and right-handed (R) relative to the longitudinal axis, independently of its neighboring protofilament (Figure 2b) [83]. During swimming, native filaments adopt a superhelical or corkscrew shape and their rotation creates thrust. Depending on the number of L- and R-protofilaments, the pitch of the superhelix changes, modulating the swimming properties [84]. If all protofilaments are in the L- or R-state, the filament is straight and cannot create a thrust and, thus, cells are immotile. Apart from handedness, L- and R-filament also differ in length, with the R-filament being 1.5% shorter. During the normal smooth swimming of *S.* Typhimurium and *E. coli*, two R- and nine L-protofilaments form a filament that is a left-handed superhelix with a pitch of around 2–3 µm. The increased number of R-protofilaments eventually results in right-handed curly flagella with a shorter pitch typical for tumbling movements [84].

Straight filaments in which all protofilaments are locked in either L- or R-state are much more amenable for structural studies than the native wavy forms in which, due to the presence of a mixture of L- and R-protofilaments, the strict helical symmetry is disturbed [85]. Several mutations of the *fliC* gene in *Salmonella* result in formation of straight filaments and were used in X-ray and EM studies [83]. Additionally, these mutations served as a basis to design the equivalent L- and R-mutants in other bacteria like *P. aeruginosa* and *C. jejuni* and to study their structures [64,86].

A cross-section of the *Salmonella* filament reveals a densely packed helical core 115 Å in diameter around a central channel that is ~25 Å wide, and an outer region exposed to the surface where domains of the individual molecules are clearly separated [87,88]. The diameter of the whole filament is ~230 Å. The core is organized into two tubes, inner and outer, connected through eleven short spokes, while the outer region contains two domains that are clearly defined.

Using cryo-EM, Yonekura et al. [62] obtained an electron density map of ~4 Å resolution that allowed them, in combination with the already known structure of FliC, to build the first complete atomic model of the R-filament from *Salmonella*. This model greatly contributed to understanding of the filament architecture, especially of its core, where the boundaries between domains of individual molecules were not obvious.

In regard to geometry, the *Salmonella* R-filament is a helical structure in which the FliC molecule is rotated 65.8° to the right followed by the translation of 4.7 Å. Protofilaments are tilted to the right by 3.5° relative to the longitudinal axis. The inner and outer tube are made of the D0 and D1 domains, respectively. In the inner tube, interactions between subunits are found along 11-start and 5-start helices and they are mostly hydrophobic, stabilizing the filament. The outer tube has a similar pattern with interactions found in the 11-start, 5-start and 16-start helices but they are predominantly polar–polar and charge–polar. Along the 11-start helix, the D1 domain of the upper subunit forms a concave surface that is complementary to the convex surface of the lower subunit and the two neighboring molecules make numerous Van der Waals contacts [61]. In the 5-start direction, the N-terminal helices of one molecule interact with the β-hairpin and C-terminal helix of the other molecule. N-terminal helices also make contact with the spoke in the 16-start direction. According to this rigid model, except for the small region of D2 proximal to the core, D2 and D3 do not engage in obvious inter-molecular contacts and they are projected away from the core. However, they do contribute to the overall stability of the filament, since deletion of the large part of this region makes the filament much more fragile in comparison to the wild type and mutant filaments, showing a contact between the adjacent outer domains along the 5-start helix that is absent in the mutant [40,89]. Therefore, it is likely that D2 and D3 exhibit flexibility in vivo, transiently interacting with the neighboring subunits and contributing to the filament integrity.

Recently, the L- and R-type filament structures of several other Gram-positive and Gram-negative bacteria became available, confirming that the 11-protofilament organization is common for all species and that the core is structurally conserved. While *B. subtilis* does not have any outer domains, D2 and D3 in *P. aeruginosa* appear as a single domain in which the D3 portion extends along the axis and seems to form a dimer with the D2 of the subunit above, although the structure could not be resolved due to low resolution in that region [86]. Another distinctive feature of the *Pseudomonas* filament is the existence of a seam along one of the protofilaments, which introduces a non-helical perturbation. This perturbation is characteristic of the so-called complex filaments that are found in some species like *Rhizobium lupini* or *Pseudomonas rhodos*, but in *P. aeruginosa* it is present only at the level of the outer domains [86]. As mentioned previously, the outer region in *Campylobacter* is significantly larger containing 3 domains and, apart from interactions along the protofilament, it also makes contacts in 5-start and 6-start directions, contributing to filament integrity [64].

### 4.1. Comparison of L- and R-Filaments

One of the questions that draws much attention is the structural basis for the polymorphic switching of the filament. Comparison of the L- and R-structures obtained by cryo-EM revealed that the D0 domains align well with some local changes in conformation, while the rest of the molecule is twisted by 5° in the counterclockwise direction [90]. Previous X-ray diffraction study on L- and R-types from *Salmonella* showed that there were no changes in helical parameters in the inner region of the core made of D0 domains [91]. However, the two types differ in the outer regions and the distance between two subunits in the protofilament is 0.8 Å shorter in the R-type compared to the L-type. Maki-Yonekura et al. [90] found that the packing of the hydrophobic side chains at the lower portion of D1 is different between L- and R-type and that the intersubunit interactions along the 5-start helix are important in stabilizing the conformation. Based on molecular dynamics simulations of the polymorphic supercoiling mechanism, Kitao et al. [92] identified three types of interaction between subunits: permanent (the same pair of residues in different states), sliding (the same types of interaction with variable partners) and switch interactions that were responsible for locking the protofilament interface in R- or L-handed state. The switch interactions were found only along the 5-start helices. However, this was not supported by the analysis of the corresponding interactions in L- and R-filaments of *B. subtilis* [86].

### 4.2. Filament Cap

Originally designated as a HAP2, FliD is localized at the tip of the filament where several copies of it assemble into a capping structure. The presence of the cap prevents the leakage of flagellin that is continuously exported through the flagellar type III secretion system into the filament channel and facilitates its insertion at the growing end of the filament. Early cryo-EM studies of the *Salmonella* cap showed that it is a stool-like pentameric structure with the flat star-shaped head region and five legs positioned almost at the right angle. The head region is 145 Å in dimeter and the height of the cap is 125 Å.

**Figure 3:**
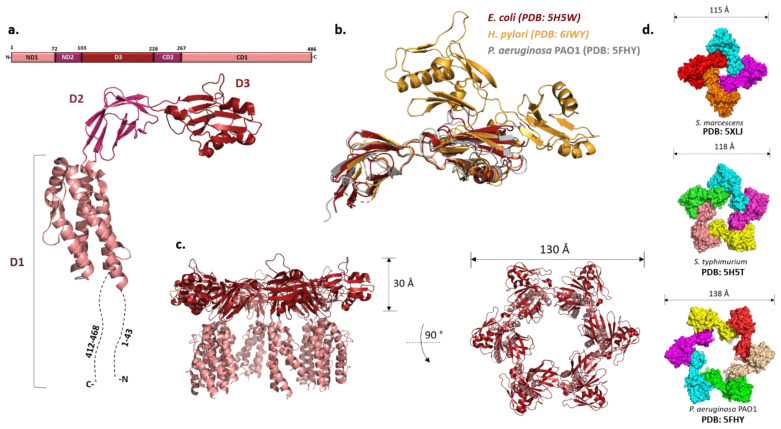
FliD and the filament cap. (**a**) Structure of FliD from *E. coli* shows three domains (D1–D3) (PDB: 5H5V). The N- and C-termini are highly flexible and could not be resolved. (**b**) Structural alignment of FliD molecules from different species show high structural homology of D2 and D3 domains. FliD from *H. pylori* has two additional domains, D4 and D5. (**c**) Crystal structure of the filament cap from *E. coli* showing a hexameric structure. Domains D2 and D3 form the head region, while D1 (incomplete) forms the flexible leg region; (**d**) Oligomeric states of the crystallized FliD caps differ across species.

While they vary in size, FliDs from different bacteria have conserved N- and C-terminal regions predicted to be coiled-coils and are largely unstructured in the monomeric state [93]. The first high-resolution structure of the *P. aeruginosa* FliD showed that this 50 kDa protein has three distinct domains: a highly flexible D1 domain that corresponds to the leg domains seen in the cap structure, and compact D2 and D3 domains that comprise the head [94]. Much as in flagellin, a continuous sequence in the middle of the polypeptide folds into D3 while domains D1 and D2 consist of N- and C-terminal portions of the chain relative to D3. Domains D2 and D3 are each made of two antiparallel β-sheets, while D1 could not be resolved with confidence apart from one helix. The structure of the *E. coli* FliD offered more insight into D1, showing that it is a helical bundle of at least four helices with a β-hairpin similar to the flagellin D1 domain, although approximately 50 terminal residues from both sides were not present in the construct (Figure 3a,c) [95]. These structures, together with the ones from *Salmonella* and *Serratia marcescens* [95,96], indicated that the structural organization of the capping proteins among different bacteria is preserved. However, not all FliDs are of the same size. For example, the FliD of *H. pylori* is 74 kDa and the crystal structure revealed that apart from domains D1–3 present in the previously studied proteins, it has two additional domains, D4 and D5, both of which reside in the head region with similar overall folds to D2 and D3 (Figure 3b) [97]. These domains may be related to the specific role FliD plays as a virulence factor during *H. pylori* infection; stronger antigenicity of D4 and D5 further supports this [97].

Once the hook and the junction are formed, FliD forms the filament cap, promoting the polymerization of FliC. The oligomeric state of the filament cap is still an open question. Initial characterization of the recombinant FliD of *Salmonella* showed the existence of decamers in solution, but the combination of cross-linking experiments, analytical ultracentrifugation and electron microscopy revealed that a pentamer is the functional unit, decamers being the product of a non-physiological cap dimerization. This was generally also accepted as the case for other bacteria, and the recent cryo-EM structure of the pentameric *C. jejuni* cap supports it [98]. However, both *Pseudomonas* and *E. coli* FliDs crystallized as hexamers and *Serratia* FliD as a tetramer (Figure 3d) [94,95,96]. Moreover, tetramers and pentamers of *Pseudomonas* FliD and tetramers of *Salmonella* FliD were observed in vitro [94,98]. While these stoichiometries could arise due to non-physiological conditions, in the case of *P. aeruginosa* hexamers were also detected in vivo [94].

The mechanism of cap-filament interaction is yet to be explained. During filament elongation, new flagellin molecules arrive at the tip continuously. This would require high mobility of the cap and continuous conformational changes of the D1 domains, most probably independently from one another, in order to accommodate arriving FliC subunits, all while staying firmly attached to the filament. One way in which FliD could interact with FliC is through its highly flexible terminal regions, present in all proteins in the outer part of the flagellum and are shown to mediate protein–protein interactions.

## 5. Protein Synthesis, Export and Filament Elongation

### 5.1. Genetic Regulation of FliC and FliD

**Figure 4:**
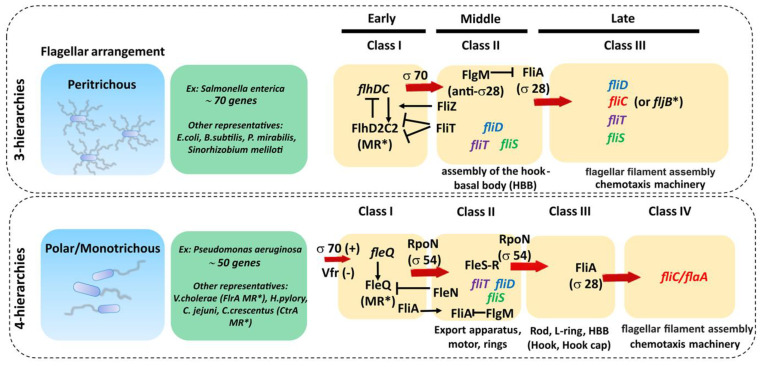
Genetic organization of main flagellar proteins involved in filament formation. The transcription of flagellar genes is hierarchical and controlled by different promoter classes that are temporally regulated. In peritrichous bacteria, flagellar genes are organized into three classes (I–III) and in monotrichous/polar bacteria the flagellar genes are organized into four classes, both starting with the transcription of the class I master regulator (MR*). Red arrows indicate the proteins and/or transcription factors responsible for the transcription activation of each corresponding class of flagellar genes. Black narrow arrows indicate positive activation/induction and black arrows with a vertical line tip (|) indicate inhibition. Genes involved in filament synthesis and their cognate chaperones are represented in italic colored font.

The genetic organization of the flagellar components is highly complex and relatively well conserved across bacteria with the same type of flagellar arrangement (i.e., peritrhichous, monotrichous/polar, etc.) but quite different between them [1,99,100]. The bacterial flagellar regulon commonly consists of dozens of genes grouped in several operons, encoding all structural proteins of the flagellum, the chemosensory apparatus and the regulators that control gene expression. For example, there are about 50 genes involved in the synthesis of the polar *P. aeruginosa* flagella, distributed in 17 putative operons comprising 41 flagellar genes (26 encoding structural proteins, eight encoding regulators and six involved in the export apparatus), linked to nine genes involved in chemotaxis and clustered in three regions of the chromosome constituting the *fla* regulon [101,102]. On the other hand, in peritrichous bacteria such as *Salmonella* spp. and *Escherichia coli*, the flagellar regulon contains 70 genes distributed in at least 25 operons [79,99]. The transcription of these genes is hierarchical and controlled by different promoter classes that are temporally regulated (Figure 4, Table 1). In peritrichous bacteria, flagellar genes are organized into three classes (I-III), starting with the transcription of the class I master operon as a response to the environmental stimuli that leads to the synthesis of the transcriptional regulator FlhDC. This regulator, in turn, activates the transcription of the class II genes responsible for the assembly of the hook-basal body (HBB) complex. Among class II genes are two crucial regulators of the class III genes: transcription factor FliA (σ^28^) responsible for the transcription of class III genes, and FlgM, which acts as a FliA inhibitor. Once the HBB is assembled, FlgM is exported out of the cell via the HBB central channel, releasing FliA to interact with RNA polymerase and guide the transcription of the class III genes, including flagellin. This three-tiered organization ensures that cells produce flagella in response to the environmental signals and that energetically costly synthesis of the filament proceeds only when enough HBBs have been formed. An exception is the *Actinoplanes missouriensis* zoospore in which there is no hierarchical coordination and 33 flagellar genes are transcribed simultaneously during the sporangium formation [103]. The filament cap gene *fliD* belongs to the group of class II genes, whereas flagellin genes belong to class III. Although *fliD* is expressed as a class II gene, it is not assembled into the growing flagellar structure until the hook and the junction proteins are expressed from class III (including flagellin genes) and incorporated into the flagellar organelle [104]. Hence, this cascade serves to control the timing of gene expression to coincide with the assembly of the flagellar apparatus and filament.

As mentioned above, *S.* Typhimurium exhibits phase variation, or alternate expression of two flagellin genes, *fliC* and *fljB*. This process is regulated by a control region upstream of *fljB* that is a subject of inversion, which orients the region forward and generates a promoter. This allows co-transcription of *fljB* and *fljA*, a transcriptional repressor that inhibits the expression of FliC [105].

Recently, Rao et al. found that flagellar gene expression in *Salmonella* is bimodal, meaning that in a population of genetically identical cells under the same conditions only a fraction of them is motile [106,107]. This bimodality is present at both the class II and class III levels and is governed by separate mechanisms. While a double negative feedback loop involving two flagellar regulatory proteins, RflP and FliZ, controls the expression of the class II genes in response to nutrient availability, class III gene expression is tuned by the secretion of FlgM and there is a minimum number of HBBs necessary for the cell to pass this checkpoint.

In bacteria with a polar flagellum, flagellar genes are transcribed in a four-tiered hierarchy, with genes encoding components of the HBB split between class II and class III (Figure 4) [101]. Gene regulation in *Pseudomonas* is more complex and involves the activation of the two-component system FleS-FleR and another sigma factor RpoN (σ^54^) to activate class III genes. In addition, unlike in *Salmonella*, FliA (σ^28^) is constitutively expressed in *Pseudomonas* independently of other flagellar genes, which makes it one of the class I genes [101].

In bacteria with multiple flagellins, such as *Helicobacter* and *Campylobacter*, expression is under the control of different sigma factors with FlaA under control of σ^28^, while FlaB is under control of σ^54^ [108,109]. Although the major flagellin is usually under the control of σ^28^, there are exceptions such as *V. cholera* and *S. oneidensis* in which the major flagellin is dependent on σ^54^, while commensal gut bacteria of *Eubacterium* and *Roseburia* species are under the control of σ^28^ and σ^43^ [110,111]. In the phylogenetically related *Butyrivibrio fibrisolvens*, transcription of one *fliC* gene is driven from two different promoters, yielding two transcripts with alternative transcription start-sites [111].

In some Gram-positive bacteria, changes in the environmental conditions such as nutrient limitation induce variations in the levels of the intracellular messengers guanosine tetra/pentaphosphate, (p)ppGpp, guanosine nucleoside triphosphate (GTP) and branched chain amino-acid pools. These variations are detected by a conserved GTP-sensing protein CodY and a global regulator CsrA that modulate flagellin expression. Cell motility can be repressed under elevated intracellular cyclic-di-GMP levels, by impeding transcription of some of the flagellum genes [112,113,114,115,116]. Indeed, the expression of *fliD* and *fliC* is repressed in phosphodiesterase 3 (PDE3) knockout mutants triggered by elevated c-di-GMP accumulation [117].

Because the presence of flagellin can be deleterious to the bacterium on account of inducing host immunity, flagellated bacteria downregulate (or turn off) flagellin expression during the host invasion to avoid host immune responses. Therefore, normal microbiota within the healthy adult mammalian gut has been shown to have overall relatively low levels of flagellin expression, while TLR5−/− mice exhibited a diversity of gut microbiome members with overexpressed flagellar genes [118,119].

Both commensal gut strains of motile *E. coli* and pathogenic *S.* Typhimurium strains strongly down-regulate their genes coding for flagellar machinery and lose their motility once inside the host [120,121]. Under environmental temperature conditions (22–30 °C), the expression of flagellar genes in the human pathogens *Listeria monocytogenes* and *Legionela pneumophila* is normal, but significantly reduced when raised to 37 °C, revealing temperature-dependent transcription mainly controlled by the protein GmaR, which acts as a protein expression thermostat [122,123]. These types of system provide a pathogen with the ability to turn off immune-stimulating antigens before they trigger adverse host defense mechanisms once inside their target host.

Regulation of flagellin expression also occurs on the post-transcriptional level by proteins that bind to the untranslated leader region of the flagellin mRNA, affecting transcript stability and/or ribosome access [124,125,126]. For instance, the RNA-binding protein CsrA of *B. burgdorferi* specifically mediates synthesis of the major flagellin by inhibiting translation initiation of its transcript. In some cases, post-transcriptional regulators repress the accumulation of flagellin when cells are defective for flagellar hook assembly [127,128].

An assembly checkpoint that prevents flagellin translation and assembly prior to hook completion was discovered in *B. subtilis*. This is governed by a homeostatic mechanism in which the flagellin protein itself is a critical regulator. In a partner switching mechanism, the flagellar assembly factor FliW binds flagellin, while a global regulator CsrA binds flagellin mRNA, repressing its translation. After completion of the hook, flagellin is secreted, and the released FliW binds CsrA, thus derepressing flagellin translation [129]. An interesting case is *A. missouriensis*, in which all flagellar genes are transcribed simultaneously during sporangium formation and there is no checkpoint mechanism in the process of flagellar gene transcription to optimize the efficiency of the flagellar assembly [103]. The post-transcriptional level of regulation between protein production and assembly could play an important role, although this remains unknown.

### 5.2. Chaperones

Accumulation and premature oligomerization of flagellar axial proteins in the cytosol could be wasteful and detrimental to the cell. For this reason, bacteria encode flagellar-specific chaperones dedicated to facilitating the assembly and export of the flagellum components mainly by protecting and/or preventing their cognate flagellar protein substrates from aggregation or avoiding premature undesired interactions of flagellar proteins in the cytoplasm prior to interaction with the export gate [130]. Due to the small diameter of the flagellar export central channel (20–30 Å), the proteins destined for incorporation into the growing flagellum must be exported in a partially or completely unfolded state, implying that premature folding and oligomerization in the cytosol must be prevented to keep them in a secretion-competent conformation for flagellum assembly [131]. Hence, the external flagellar components require T3SS-specific chaperones to facilitate their efficient export [99].

#### 5.2.1. FliS—Flagellin Chaperone

FliS acts as a flagellin-specific T3SS chaperone of FliC, preventing premature folding and inappropriate interaction of newly synthesized flagellin subunits in the cytosol, thus facilitating its export and polymerization upon completion of the HBB assembly. Yeast two-hybrid assays indicated that the C-terminal disordered region of flagellin is essential for FliS binding and, accordingly, spontaneous mutations causing flagellin accumulation in the cytoplasm map to the C-terminal region of FliC [132]. Thermodynamic experiments indicated that FliS does not function as an anti-folding factor keeping flagellin in a secretion-competent conformation. Instead, FliS binding stabilizes the flagellin conformation through formation of the α-helical secondary structure in the last 40 C-terminal residues of FliC (residues 454–494) [105,131,133].

The first crystal structure of a flagellar chaperone, *Aquifex aeolicus* FliS (AaFliS), revealed a novel, mainly α-helical fold, different from those of the T3SS chaperones [134]. The structure of *Aa*FliS chaperone in complex with a C-terminal fragment of its cognate flagellin (PDB: 1ORY) shows a monomer-to-monomer interaction. However, it was also reported that FliS binds in a 2:1 stoichiometry to the C-terminal region of flagellin to completely prevent the premature polymerization of newly synthesized flagellin molecules, as a 1:1 FliS to FliC molar ratio was able to prevent FliC polymerization only partially. Intriguingly, no stable FliS–FliC complex could be detected in *S. typhimurium* cell extracts subjected to gel-filtration chromatography, suggesting that this interaction may be transient in vivo. Such rapid chaperone dissociation would favor the subsequent export of FliC [135].

**Figure 5:**
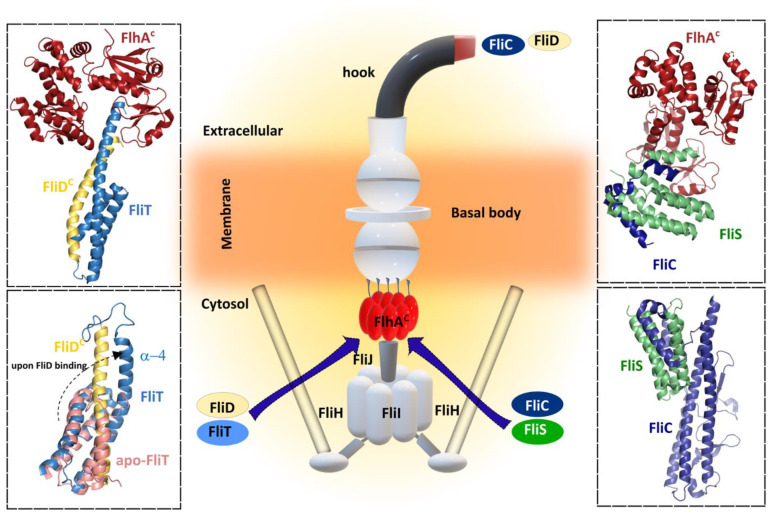
Bacterial flagellar filament proteins and their cognate specific chaperone form complexes with FlhA for filament exportation/synthesis. FliC and FliD proteins interact with each cognate chaperone FliS and FliT, respectively, before interacting with the cytosolic region of FlhA (FlhA^C^). **Upper left**—Cartoon representation of crystal structure 6CH2 (FlhAC-FliT-FliD complex from *Salmonella enterica* subsp. *enterica* serovar Typhimurium str. LT2. **Upper right**—Cartoon representation of crystal structure 6CH3 (FlhAC-FliS-FliC complex) from *Salmonella enterica* subsp. *enterica* serovar Typhimurium str. LT2). **Lower left**—Superposition of cartoon representation of both NMR solution structures: 5KRW (FliD-FliT) and 5KS6 (apo-FliT) from *Salmonella enterica* subsp. *enterica* serovar Typhimurium str. LT2. The FliT helix α4 is released upon FliD binding. **Lower right**—Cartoon representation of crystal structure 5MAW (FliS-FliC complex) from *B. subtilis*. **Center figure**—simplified schematic representation of the main flagellar components.

The interaction between FliS chaperone and FlhA is a key step preceding the efficient transfer of FliC to the platform of the flagellar type III export apparatus for a rapid export during flagellar filament assembly [136,137,138]. Filament proteins in complex with their cognate chaperones bind to a highly conserved hydrophobic pocket of FlhA^C^ to promote unfolding and protein translocation by the protein export apparatus. It was predicted that different binding affinities of FlhA^C^ for the chaperone substrate complexes may be the key to defining the correct order of protein export among the filament-type proteins [139]. Recently, a structure of the ternary complex formed by FliC, FliS and the export gate protein FlhA revealed that FliC does not interact directly with FlhA (Figure 5). Instead, the presence of FliC induces a binding-competent conformation of FliS that exposes the motif which is specifically recognized by FlhA [140]. Moreover, SAXS and HDX-MS experiments showed the formation of a heterotrimeric FliC-FliS-FliW complex that interacts with FlhA suggesting that FliS and FliW are released during flagellin export. FliW and FliS bind to opposing interfaces located at the N- and C-termini of flagellin, respectively, and these proteins seems to synchronize the production of flagellin with the capacity of the T3SS to secrete flagellin [141].

FliS plays an additional role in suppressing the secretion of FlgM in *Salmonella*. The loss of FliS results in a short filament phenotype despite high expression levels of FliC, which is explained by the increase in the secretion level of FlgM [136]. Bypass mutants have been isolated from a *Salmonella ΔfliS* mutant, and all those mutations were identified in FliC [142,143].

Galeva et al. and Xu et al. investigated the direct interaction between FliS and FlgM from *S. typhimurium* and *Yersinia pseudotuberculosis*, respectively [135,144]. Using a number of different approaches, they showed that these proteins specifically interact to form a 1:1 complex, and that this interaction protects FlgM from proteolysis. FliS acts as a negative inhibitor of FlgM secretion, keeping this intrinsically disordered protein stable before FliA is expressed in cells. The binding site of FliA on FlgM is close to or even overlaps with the binding site of FliS, suggesting that FliA binding removes FliS from the complex. In addition, FliS from *S. typhimurium* is expressed from both class II and class III promoters, while FliC is expressed only from a class III promoter, indicating that FliS is synthesized and stabilized by FlgM prior to FliC (or FljB) production.

#### 5.2.2. FliT—Chaperone of FliD

For the correct formation of the filament, a filament-capping protein FliD should be exported first during the filament assembly to form a penta- or hexameric cap that promotes self-assembly of FliC. In order to do this, FliD requires the assistance of its chaperon FliT [145]. FliT act as key flagellar chaperone in the assembly and operation of the flagellum because it binds to several flagellar proteins in the cytoplasm, such as the export apparatus components FliI, FliJ, and FlhA, beyond interaction with its cognate FliD. As an example of its versatility, FliT also functions as a negative transcriptional regulator of flagellar genes by inhibiting the formation of a DNA complex with the master regulator FlhDC. Lately, several crystal structures of FliT alone and in complex with FliD or FliI have become available [130,145]. Although in the crystal structure of *Salmonella* FliT was present as a tetramer, the observation that FliT was in the monomer–dimer equilibrium under physiological conditions suggests that the tetrameric form was an artefact of crystal packing. The structure revealed, however, that FliT adopts an antiparallel four α-helix bundle and uses a hydrophobic surface formed by the first three helices to recognize its substrate proteins. In the absence of a substrate protein, FliT adopts an auto-inhibited structure conformation in which both the binding site for the partner proteins (the hydrophobic surface formed by helices α1–α3) and the binding site for FlhA (helix α4) are occluded. This auto-inhibited structure may serve to protect the substate-binding surface from aggregation in the absence of substrates and to mask the binding site for FlhA, adopting an uninhibited conformation only when the substrate protein is to be targeted to the export gate. The auto-inhibition/activation and targeting mechanism reported for FliT appears to be shared among other flagellar chaperones such as FliS, which also adopts an auto-inhibited structure, released upon FliC binding. The N-terminus of FliS serves as the FlhA binding site, and in these chaperones a highly conserved Tyr residue appears to be essential for efficient FlhA binding, suggesting that these chaperones may use similar strategies for substrate binding and activation of the complexes for binding to FlhA and thus for targeting to the export gate. A recent structure of the FlhA−FliT−FliD ternary complex revealed that there is no direct interaction between FliD and FlhA, as was observed with FlhA–FliS–FliC ternary complex (Figure 5) [140].

Although the structural architecture of FliT is similar to that of FliS, the arrangement of α-helices is different. FliS forms a heterodimer with its cognate substrate FliC. The C-terminal region of FliC interacts with all three α-helices of FliS in an extended conformation. Similarly, FliT forms a stable heterodimeric complex with FliD in solution through an interaction between the C-terminal half of FliT and the C-terminal region of FliD, similar to that of the FliS–FliC complex. However, only the highly conserved surface-exposed Lys79 in α3, is critical for the interaction with FliD suggesting that the C-terminal region of FliD might interact with α3 of FliT in a different manner from the FliS–FliC interaction. Database searches revealed that FliS is the most widely conserved and ancient flagellar chaperone and it has been proposed that other flagellar chaperones, such as FlgN and FliT, has evolved from FliS. Biochemical and structural analyses have provided insight into how the C-terminus of FliT regulates its interactions with the FlhDC complex, FliI ATPase, and FliJ (subunits of the export apparatus that may function as a general chaperone), and the conformational change that is responsible for the switch between binding partners during flagellar protein export. After completion of HBB assembly, the C-terminal α4 helix of FliT is released from the hydrophobic cleft formed by the α2 and α3 helices to allow the FliT–FliD complex to bind to the FliH–FliI–FliJ complex through the specific interactions of FliT with FliI and FliJ, and the entire complex binds to the docking platform of the export gate. After FliD is unfolded and translocated into the flagellar channel to assemble the filament-capping structure at the tip, unbound FliT is free to interact with FlhC and form a complex FliT–FlhD4C2, thereby suppressing the class II gene expression, functioning as an anti-FlhD4C2 factor. FliT later dissociates from the FlhDC complex, allowing the free FlhDC complex to activate the transcription from the class II promoters anew.

Kanhra et al. proposed a mechanistic model of how the versatile FliT chaperone may assist with the assembly and complete formation of the flagellum [130]. In this sequentially organized model, FliT binds to FliJ to deposit it at the export gate via interaction between the FliT α4 helix and FlhA. Once FliJ is anchored to the export gate, FliT transports FliI at the membrane, helped by FliH that remove FliI from FliT, and FliI forms a hexamer around FliJ. Finally, FliT binds to FliD and delivers it to the membrane for FliD exportation.

The molecular insight into the interactions of FliC and FliD with their cognate chaperones FliS and FliT provides a picture of well-organized and temporally regulated interactions that orchestrate the flagellar filament assembly, although the possibility of discovering a missing player/step in this complex process in the future cannot be discarded.

### 5.3. Flagellin Export and Assembly into the Growing Filament

The export of flagellar proteins is a highly organized and well-controlled process conducted by the flagellar T3SS apparatus located at the base of the flagellum, and powered by ATP and proton motive force (PMF) across the cytoplasmic membrane as the energy sources [99,136,146,147,148]. The T3SS pumps unfolded flagellin monomers into the flagellum’s central channel together with its cognate filament cap FliD, which are necessary for in vivo filament elongation. T3SS also transports anti-sigma factor FlgM into the culture media, upon completion of the basal body assembly, allowing σ^28^ to transcribe flagellar filament genes [149,150].

What constitutes the driving force for flagellin assembly in the growing filament remains an open question, considering that this process occurs far away from the cell where no conventional energy source is available. A possible answer was suggested by Evans and coworkers, when they proposed a model of filament growth consisting of an inter-subunit chain mechanism in which the growth would be powered by the flagellin subunits themselves, since they would be linked head-to-tail, forming a chain that is pulled across the entire filament to the tip [151,152]. According to this model, each newly linked unfolded flagellin subunit would be moved up from the gate into the filament pore by the entropic pulling force of the folded subunit anchored at its other end at the flagellum tip. In this way, successive rounds of subunit linking at the cell membrane export machinery would be coupled to the subunit folding at the tip to allow a continuous intrinsically energized subunit transit, resulting in a constant rate growth of the flagellum independent of the filament length [152,153,154]. However, this model has certain incompatibilities with the known biophysical properties of flagellum assembly [61,62,155]. In contrast to this model, Renault et al. recently shed a light on the dynamic self-assembly process of filament growth, using in situ labelling and real-time immunostaining of elongating individual flagellar filaments [156]. They demonstrated that flagellar growth outside of the cell is controlled by a simple injection–diffusion mechanism. In this model, the flagellin monomers, which are at least partially α-helical inside the channel, are pushed by a PMF-driven export apparatus into the flagellar channel and spread along the way passively by diffusion in one dimension [154,157]. Hence, this new molecular mechanism proposed for bacterial flagella growth, provides a simple explanation to why the flagellar filament does not grow infinitely in the absence of any other length-control mechanism in place. They calculated that flagellar filament growth rate (initially ~1700 amino acids per second) decays exponentially depending on filament length so, accordingly, the longer the flagellum gets, the slower it grows, as proposed initially by theoretical modeling and molecular dynamics simulations [155]. In addition, the inhibition of the PMF-dependent export apparatus revealed a major contribution of substrate injection in driving filament elongation. Although injection-diffusion is currently the most plausible model, further studies are needed to understand how the type III secretion system harnesses chemical energy to drive the movement of flagellin far outside the cell into the growing flagellum, and how the flagellin/channel friction interactions modulate the rate of flagellin monomers translocation. In this regard, recent analysis of the flagellin structure of *S. Typhimurium* (PDB ID: 1UCU) indicated that the hydrophilic inner surface of the narrow internal flagellar channel is lined with solvent-exposed side chains of charged and polar residues from the C-terminal D0 domain of FliC (residues Q^484^, N^488^, S^491^ and R^494^) [158]. It was recently proposed that this hydrophilic inner surface of the channel should minimize retention of unfolded protein monomers to facilitate their rapid diffusion to the distal end of the filament for assembly. However, these flagellar channel-lining residues were not strictly required for flagellin export and assembly, although substitutions of these residues affected the stability and morphology of the flagellar filament [158].

## 6. Filament–Host Interaction

**Figure 6:**
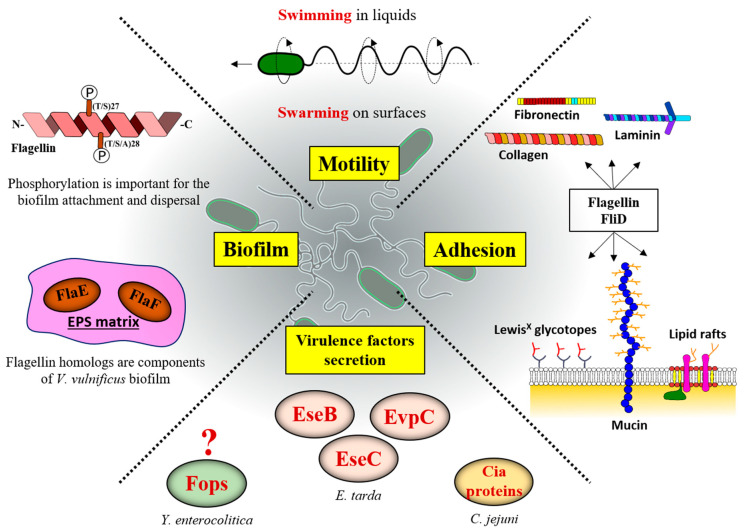
Flagellar filament, flagellin and FliD are implicated in various processes.

In addition to being an organelle of locomotion, the versatility of the bacterial flagellum includes the ability to adhere to a variety of substrates, to aid in the formation of biofilms in nature, to function as a secretory machine that can export proteins promoting diverse biological processes, and to mediate inflammation in both plants and animals (Figure 6) [3,159,160].

### 6.1. Filaments Are Directly Involved in Surface Adhesion

Flagella-mediated adhesion to plasma membranes is a crucial step that allows bacteria to penetrate and colonize the mucus layers of their hosts and invade the tissues [159]. The adherence of *C. jejuni* to intestinal cells is dependent on flagella in combination with LPS, while in *Aeromonas caviae* cells without polar flagellum were almost completely nonadherent [161,162]. Flagellin and/or flagellar cap proteins have been identified as adhesins in other pathogens such as *E. coli*, *C. difficile*, and *P. aeruginosa*.

Mucin is an important target for *P. aeruginosa* adhesion as the lung epithelium contains a thick mucus layer. In the *P. aeruginosa* PAK strain, FliD appears to mediate the adhesion to mucins and this is not due to its effect on filament formation, since the mutant in which FliC was abrogated still binds mucins [163,164]. Conversely, in the PAO1 strain FliC participates in mucin adhesion [165]. Moreover, the same study indicated that FliC and FliD of PAO1 bind Lewis^X^ glycotopes which are typically found on the airway mucins, while FliD from the PAK strain does not [165]. In the case of binding to Muc1, however, it appears that deleting *fliC* in the PAK strain abolishes adhesion, while deletion of *fliD* has no effect [166]. This finding is surprising considering that FliD is necessary for filament formation. In addition, PAK-Δ*fliC* is impaired in adhesion to human airway epithelial cells and exhibits reduced virulence in a mouse model of pneumonia.

Similarly, *E. coli* flagellin is also important for adhesion, as in enteropathogenic *E. coli* (EPEC), or in enterotoxigenic *E. coli* (ETEC), where it works in concert with an exoprotein adhesin EtpA by capturing and presenting it to the eukaryotic receptors [167,168]. Among *E. coli* strains, the differences in flagellin determine their ability to adhere to specific surfaces. For example, the FliC of enterohaemorrhagic *E. coli* (EHEC), but not of EPEC, provides specific binding to the bovine terminal rectal epithelium and agglutinates rabbit red blood cells, while EPEC FliC binds to collagen, and to a lesser degree laminin and fibronectin [169,170]. Not only pathogenic, but also probiotic, *E. coli* possess adhesive flagella that are important for gut colonization. Such is the case for the *E. coli* Nissle 1917 strain in which deletion of *fliC* abolishes the adhesion to the intestinal epithelium, an interaction that is mediated through the mucus component gluconate [171]. On the other hand, when it comes to the direct role of flagellin and FliD in adhesion in *C. difficile* and *H. pylori*, reports are somewhat contradictory in the literature, with some studies showing their direct involvement in different cell types [172,173], while others suggest that their importance is limited to providing motility while other adhesins mediate binding to colonize the gastric epithelial cells [159,174].

Membrane lipid composition and physical properties of the lipid bilayer, such as membrane fluidity and head-group lipid packing, have been shown to be key parameters that enable flagellar adhesion. Saturated fatty acids provide optimal binding of flagella, while polyunsaturated fatty acids prevent bacterial adhesion on membrane bilayers and play a key role for optimal host colonization [175]. It has been observed that flagella preferentially bind to cholesterol and sphingolipid-enriched lipid microdomains and that flagellin is required for adhesion to lipid rafts in host cells. Indeed, *S. typhimurium* flagella have been shown to interact with a cholesterol-coated surface; adhesion was significantly reduced in Δ*fli*C mutant [176].

### 6.2. Flagellar Filament in Biofilm Formation

The involvement of flagella in biofilm formation is well-documented and many species in which *fli*C was deleted exhibit significantly reduced biofilm formation on diverse surfaces, suggesting that flagellin *per se* plays a role in biofilm formation [160,177,178,179,180,181]. Besides *fliC* involvement, studies in *Cronobacter sakazakii* strain ES5, an opportunistic food-borne pathogen, showed that biofilm formation was drastically reduced in *fli*D and *flh*E mutants, suggesting a direct interaction of flagellar filament and Caco-2 cells [182].

Beyond the necessity of filaments for bacterial attachment, they may affect biofilm formation through certain specific functions. In the *P. aeruginosa* PAO1 strain, the absence of phosphorylation at the conserved T27 and S28 residues of FliC does not affect swimming motility, but it does affect the secretion levels of extracellular proteases by the type 2 secretion system (T2SS) and biofilm formation [183]. Residues T27 and T28 are conserved in the N-terminal domain of flagellin across *Pseudomonas* sp., *Escherichia* sp. and *Salmonella* sp. with the canonical sequence being (T/S)27, (T/S/A)28, implying that flagellin might have some function other than motility, where these phosphorylation sites could play a role. The effects of FliC phosphorylation on biofilm attachment and dispersal indicate that initial attachment and detachment during the dispersal stage are delayed by the loss of FliC phosphorylation in static and dynamic flow biofilms. As each of these processes still proceeded in the absence of phosphorylation, it was suggested that FliC phosphorylation regulates the timing and rate of these processes without affecting biofilm architecture. The FliC-T2SS interaction is evident during biofilm growth of *P. aeruginosa*. Taken together, these data suggest that FliC phosphorylation could act to integrate environmental cues with the signals for attachment or dispersal of biofilms playing a key role in ecological adaptation of this opportunistic environmental pathogen [183].

Polar flagella of *Vibrio* contain multiple flagellin subunits and, in addition to the four that build the filament, two flagellin-homologous proteins (FHPs), FlaE and FlaF, are also present. Neither FlaE nor FlaF, however, are involved in the construction of flagellar filaments and cellular motility. Recently, Jung et al. demonstrated that *V. vulnificus* mutants defective in both *fla*E and *fla*F genes significantly decreased biofilm production compared to wild-type strains, revealing that FlaEF are involved in strengthening the exopolysaccharide (EPS)-enriched biofilm matrix, thus being essential dominant constituents and crucial in the maturation of biofilms [184]. Both proteins are well expressed and secreted into the extracellular milieu through the secretion apparatus for flagellar assembly and significant levels of FlaEF were detected in the extrapolymeric matrix of *V. vulnificus* biofilm. Although FHPs pass through the same channel as flagellin subunits, FHPs exhibited significantly lower affinities for HAPs, such as FliD and FlgL, and it was assumed that these FHPs are just excreted to extracellular milieu without being assembled into a flagellar structure and without any interaction with flagellins. Other pathogenic *Vibrio* species also exhibited the presence of FHPs that are involved in biofilm enhancement, which, in turn, may facilitate increased pathogenicity of these bacterial species [184].

### 6.3. Secretion of Non-Flagellar Proteins during Host Invasion

Flagella are commonly recognized as important virulence determinants of bacterial pathogens. The type III protein secretion of the flagellar system may be a general mechanism for the transport of proteins that influence bacterial–host interactions. The flagellar apparatus can secrete several putative virulence proteins, effectors, or toxins, having a wide range of functions, including cytotoxicity, hemolysis, proteolysis and protein phosphorylation/dephosphorylation [185]. Early studies of the pathogenic bacterium *Yersinia enterocolitica* revealed that its flagellar T3SS export apparatus promotes secretion of non-effector and non-flagellar extracellular proteins termed Fops [185]. One of the Fops proteins exported by the flagellar secretion system was the virulence-associated phospholipase, YplA, and its secretion is required for full virulence of *Y. enterocolitica* [185,186]. Further studies revealed that YplA was secreted by two endogenous injectisome T3SSs in *Y. enterocolitica* and that filamentless mutants were still competent for YplA secretion, indicating that filament is not essential for the secretion of this phospholipase [187,188].

The filament also affects secretion of virulence factors in *Edwardsiella tarda*, a flagellated and virulent Gram-negative bacterium that causes edwardsiellosis in fish. In a *fliC* in-frame deletion mutant, the diameter of the flagellar filaments is significantly reduced and this mutant shows 100-fold reduced pathogenicity to fish. FliC is required for the protein secretion of the T3SS and T6SS in *E. tarda*, since the Δ*fliC* mutant exhibited a significant diminution or complete absence of three effector proteins secreted though either the T3SS (EseB and EseC) and or the T6SS (EvpC) [181].

*C. jejuni* flagellum has also been implicated as a secretory machine for proteins not required for motility, such as the *Campylobacter* invasion antigens (Cia proteins). These proteins are required for *C. jejuni* invasion of human intestinal epithelial cells or survival within these cells [189,190,191,192]. Early studies found that inactivation of the flagellar *flaAflaB* locus in *C. jejuni* strain 81,116 abolished secretion of the Cia proteins [193]. However, a more recent paper showed through a different mutagenesis method that, when the *fla*A*fla*B locus was completely deleted, the mutant strains were still able to secrete the Cia proteins, demonstrating that FlaA and FlaB are not required for secretion of the Cia proteins [194]. Only a minimal flagellar structure that includes the hook and hook-filament junction seems to be required for secretion of the Cias [194]. Thus, the flagellar cap protein FliD and filament proteins FlaA and FlaB are dispensable for Cia protein secretion, while the hook protein FlgE and hook-filament junction proteins FlgK and FlgL are required. These authors suggested that the flagellar T3SS without a filament could function analogously to an injectisome. In addition, there may be additional proteins that interact with the flagellar hook (i.e., FlgE, FlgK, and/or FlgL) to enable direct delivery of CiaC, that interacts with the tip of the injectisome needle. *C. jejuni* flagella are involved in the secretion of FlaC, a protein found to increase *C. jejuni* invasion of Hep-2 epithelial cells [195]. Despite the N- and C-terminal regions of FlaC being highly homologous to flagellins FlaA and FlaB, FlaC lacks the central domain and is not required for the expression of a functional flagellum. At least 18 different Cia proteins have been proposed to be secreted and some of those have been functionally characterized [189,190,191,194,196,197]. The process of flagellar biogenesis and polymerization of the flagellar filament is likely to temporally influence the dynamics of secretion of virulence factors in *C. jejuni*. Usually, the secretion of virulence factors in *C. jejuni* occurs during the hook biogenesis with filament polymerization itself reducing secretion of these factors. For instance, FedB, CiaI, and FspA1 proteins are secreted during hook biogenesis, but the level of secretion of these proteins is reduced once filament polymerization begins. In addition, FedB, CiaI, and FspA1 are produced simultaneously with flagellin FlaA, but transcribed from different promoters and produced at lower levels than FlaA [198]. Thus, they may require a distinct mechanism to efficiently compete with abundant levels of flagellins for interactions with the flagellar T3SS that result in secretion. Flagellins and other flagellar proteins require a specific motif within the N-terminus for T3SS recognition [152,199,200]. Specific chaperones and intramolecular domains within non-flagellar exported proteins may assist these proteins to compete with flagellins for recognition and secretion by the T3SS. These proteins may have adopted traits common to natural substrates such as flagellin proteins to be delivered via the same secretion machinery. Many interesting questions arise about the mechanisms by which these non-flagellar proteins, not involved in motility, are specifically and efficiently secreted by the flagellar organelle. For instance, it would be important to analyze whether the secretion of non-flagellar proteins is common among flagellated bacteria and whether there are specific chaperones in charge of their export.

### 6.4. Host Immune Response to Flagella

**Figure 7:**
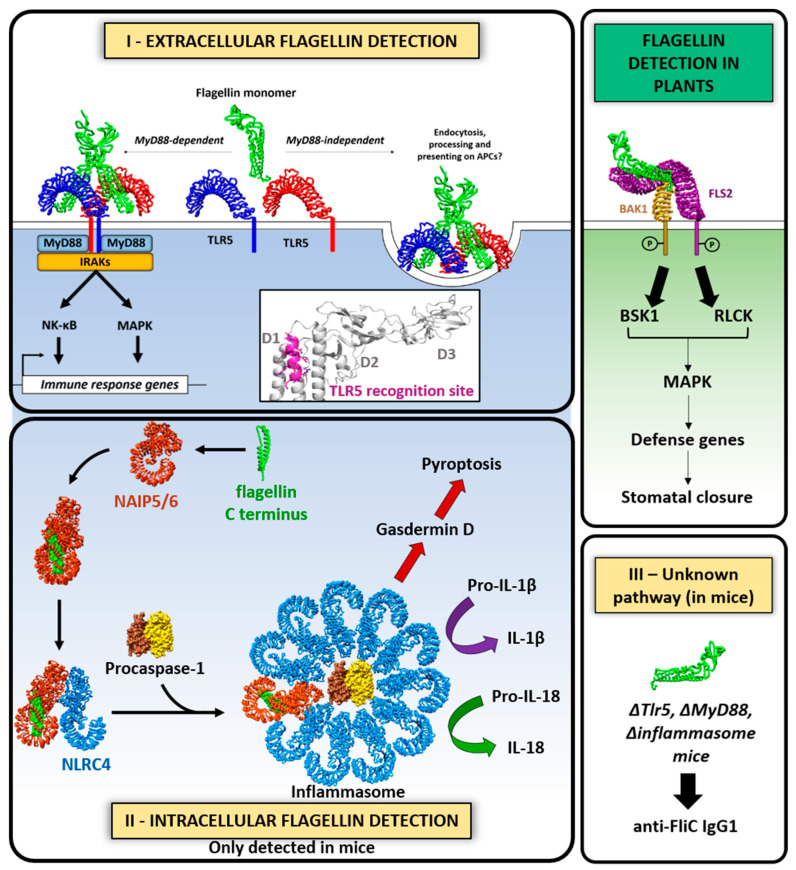
Flagellin recognition by the host. Extracellular detection—TLR5 receptor recognizes region on the D1 domain of flagellin and upon binding, TLR5 dimerizes starting a signaling cascade in the cytoplasm that leads to the alteration of gene expression; Intracellular detection (detected in mice only)—Naip 5 and 6 (NOD-like recpetors) rocognize C terminus of the flagellin. They recrute another NOD-like receptor NLRC4, which oligomerizes and activates caspase 1 forming a structure called inflammasome. Inflammasome activates molecules such as interleukins and gasdermin D leading to pyroptosis; Detection in plants—Plant receptor FLS2 recognizes N-terminal fragment of flagellin and upon binding FLS2 dimerizes with BAK1, protein kinase, starting a downstream signaling cascade that leads to the stomatal closure.

Flagella elicit the activation of host inflammatory responses via flagellin interaction with specific molecules through a variety of signaling pathways. Flagellins act as potent agonists of the innate immune system inducing proinflammatory responses, given their ability to stimulate the extracellular Toll-like receptor 5 (TLR5), the intracellular NOD-like receptor (NLR) family 4 (NLRC4)-NAIP 5/6 inflammasome, and an unknown additional third pathway recently proposed that is independent of TLR5, Casp1/11, and MyD88 (Figure 7) [201,202,203].

TLR5 features 20 tandem copies of leucine-rich repeats (LRRs) in the ectodomain, which are responsible for flagellin binding [204]. It is expressed by epithelial cells, neutrophils, monocytes, and dendritic cells [205]. TLR5 signaling triggers the transient production of immune mediators by myeloid and epithelial cells. Upon flagellin binding, TLR5 dimerizes and recruits the universal TLR-specific adapter molecule MyD88. They assemble a highly organized scaffold that acts as a signaling complex known as the Myddosome that triggers the activation of downstream molecules such as nuclear factor-ĸB NK-ĸB, mitogen-activated protein kinase (MAPK), and interferon (IFN) regulatory pathways, which turns on the transcription of genes involved in innate and adaptive immunity [206]. In human cells, low concentrations of flagellin (ED_50_ in the pM range) from *S.* Typhimurium, enteropathogenic and uropathogenic *E. coli*, *S. enterica* serotype Dublin, *S. enteritidis*, *P. aeruginosa*, *L. pneumophila*, *Listeria monocytogenes* and *Clostridium difficile*, were shown to trigger proinflammatory signaling in sentinel cells such as macrophages, monocytes, DCs, and intestinal epithelium cells. Several published studies support the requirement for TLR5 signaling via MyD88 to induce T cell-dependent antibody responses toward flagellin (reviewed in [207]). Flagellins can also directly activate maturation of dendritic cells (DC) resulting in upregulation of functions related to antigen presentation, co-stimulation, and polarization of T-helper (Th) cell responses, thereby promoting the adaptive immunity response. Gerwitz et al. demonstrated that the flagellin–TLR5 interaction is required for efficient containment of an immune challenge, with consequences for metabolic homeostasis [208]. For instance, they showed that TLR5 knockout mice developed severe intestinal inflammation (colitis). They had an altered gut microbiota profile, which is characterized by changes in the abundance of bacterial species, including elevated levels of proteobacteria, especially enterobacterial species. Flagella-related genes of commensal microbes are upregulated in the gut of TLR5−/− mice because of reduced levels of anti-flagellin antibodies in the gut [119]. In contrast to the TLR5 knockout mice, MyD88 knockout mice, with broad deficiency in TLR signaling, lacked evidence of inflammation, suggesting that the inflammation observed in the absence of TLR5 may be caused by stimulation of a compensatory mechanism, in which other TLR signaling pathways are upregulated, ultimately leading to increased inflammation. In addition, TLR5 enhances anti-flagellin T cell-dependent antibody responses in a MyD88-independent manner, suggesting that TLR5 also functions as an endocytic receptor to enhance flagellin processing and presentation by dendritic cells [209].

The TLR5 consensus target sequence is composed of residues 88–98 (LQRIRELAVQA) and located in a conserved site within D1 domain of FliC of β- and γ-proteobacteria. In addition, the C-terminal domain D0 is necessary for TLR5 signaling, as demonstrated by functional studies, as well as the N-terminal D1 motifs (82–101, 110–118) and the C-terminal D1 motif (412–438) that are important for specific interaction of flagellin with TLR5 [210]. In contrast, some important human pathogens such as *H. pylori*, *C. jejuni* and *Bartonella bacilliformis* species have developed the capacity to produce flagellins with a modified motif (DTVKVKAT, DTIKTKAT or DDIQKSMV, respectively) that cannot be recognized by TLR5 [211]. Their unique flagellin sequences contain amino acid substitutions in the TLR5 recognition site that permit TLR5 evasion, as well as compensatory mutations that preserve bacterial motility. Interestingly, cell signaling assays and structure-based analysis demonstrated that the replacement of the motif of the best-characterized flagellin FliC from *S. Typhimurium* (QRVRELAV) by the corresponding motif from *H. pylori* abolishes the interaction with TLR5. Two of the scarce flagellated commensal gut bacteria genera, *Eubacterium* and *Roseburia*, contain flagellin proteins with conserved residues (L87, Q88, R89, E92, L93, and Q96) that are found in pro-inflammatory flagellins of the β- and γ-proteobacteria, and which are critical for TLR5 signaling and flagellin polymerization [111]. When two human intestinal epithelial cell lines (T84 and HT-29) were exposed to flagellins isolated from some *Roseburia inulinivorans* and *Eubacterium rectale*, they exhibited increased secretion of pro-inflammatory interleukin-8 (IL-8) [111]. Hence, flagellin-specific immune responses should modulate the microbiome’s production of flagellin to help to maintain mucosal barrier integrity and gut homeostasis [119].

The structure of the complex between zebrafish TLR5 and FliC from *S.* Dublin showed that the contact area is extensive and involves several critical sites [212]. Two regions of FliC were essential for primary interaction with TLR5 and the establishment of TLR5-FliC 1:1 heterodimers: the C-terminal α-helix of FliC D1 domain, which is in close contact with leucine-rich repeats 1–6 (LRR1-6), and the motif comprised of residues 89–96 of FliC D1 domain that binds to LRR7–10. Dimerization of heterodimers through LRR12–13 of TLR5 results in a functional 2:2 complex. The TLR5-activating main motif (89–96) is buried deep within the filament in the flagellum core (not exposed in the intact flagella) and only flagellin monomers are able to induce TLR5 signaling, while filaments do not. Furthermore, proper folding and three-dimensional structure are critical for TLR5 recognition of flagellin and must be taken into account when designing flagellin-based adjuvants for vaccines.

Bacterial flagellin can also be recognized by the innate immune system in plants, through a pattern recognition receptor (PRR) which is analogous to TLR5 from animals (Figure 7). By screening *Arabidopsis* mutants, a receptor that binds a highly conserved 22-residue peptide derived from the flagellin N-terminus (flg22) was identified [213]. This receptor, Flagellin-sensitive 2 (FLS2), contains 28 copies of LRRs in its ectodomain (ECD) connected via a single transmembrane region to a cytoplasmic Ser/Thr kinase domain that is absent in animal TLRs [214]. FLS2 mutants are insensitive to flg22 treatment, indicating that FLS2 is essential and specific for flagellin-induced immunity. The crystal structure of the FLS2 ectodomain and the flg22 ligand revealed that LRRs 9–15 of FLS2 contribute to flg22 responsiveness [215]. FLS2 is a homodimer and upon flg22 binding associates with another receptor kinase, BAK1, through their ectodomains, triggering a signaling cascade that leads to stomatal closure [216]. Binding of flg22 also induces FLS2 internalization and degradation, serving as a negative regulation mechanism. FLS2 exhibits different ligand specificities among plant species. In tomato plants, for example, a shorter version of the flagellin epitope with 15 amino acids is enough to elicit response, but not in *Arabidopsis* and *Nicotiana benthamiana* [216].

Intracellularly, the presence of flagellin is detected through NOD-like receptors (NLR) in mice (Figure 7). Two of these, NAIP5 and NAIP6, specifically recognize the C-terminal part of the flagellin D0 domain. Upon flagellin binding, NAIP5 associates with NLRC4 to form an inflammasome that activates caspase-1. Caspase-1, in turn, induces the inflammatory response by cleaving and activating interleukins IL-1β and IL-18, and triggers the lytic form of cell death called pyroptosis. This mechanism, however, was not registered in humans, in which flagellin was not able to activate the only human NAIP molecule. The structure of the flagellin-NAIP5-NLRC4 inflammasome complex shows that NAIP5 alone binds flagellin, making numerous contacts with both helices of the D0 domain [217]. Six different regions of NAIP5 (N-terminal helix, BIR1, HD1, HD2, ID, and LRR) form a flagellin-binding pocket and confer ligand specificity. Based on two-hybrid assays, flagellins from five pathogenic bacteria (*L. pneumophila*, *S. typhimurium*, *Y. enterocolitica*, *Photorhabdus luminescens* and *P. aeruginosa*) showed a positive result while those from five others including EPEC, EHEC, *Shigella flexneri*, *Chromobacterium violaceum* and *Burkholderia thailandensis* were unable to interact with NAIP5. Additionally, those flagellins that interacted with NAIP5 also induced inflammasome activation in bone marrow-derived macrophages. These studies confirmed that the ability of flagellins to bind NAIP5 correlates with their ability to induce an immune response, and that NLRC4 acts as an adaptor through which inflammasome activation signals generated from different NAIP receptors are transduced to caspase-1 [202].

It was recently revealed that FliC is capable of inducing potent IgG1 anti-FliC responses in the absence of TLR5, the inflammasome, and MyD88. Based on this, a third pathway that promotes anti-FliC antibodies was proposed, independent of TLR5 and inflammasome, and this requires the presence of all four domains of FliC. Nempont et al. described different FliC deletions that had reduced immunogenicity, which indicated that the D2/D3 domains may be the most critical for MyD88-independent IgG1 anti-flagellin responses [218]. Mice immunized with FliC and different truncated versions made antibodies that showed reactivity to the D0/D1 and D2/D3 domains, indicating that both components of the molecule are antigenic. The D3 domain of FliC influences immunogenicity independently of the known innate recognition sites in the D0/D1 domains to augment antibody production, suggesting that full-length FliC and the preservation of all four domains would be critical for optimal immunogenicity and primary and secondary antibody responses in flagellin-based vaccines [203]. The nature of the third pathway is poorly understood and requires further investigation.

### 6.5. Flagellin in Vaccines

Due to their ability to modulate innate immunity, flagellins are increasingly considered as a potential adjuvant in vaccines [207,219,220,221,222]. Several vaccines using flagellin as adjuvant are in preclinical trials, including vaccines against influenza, malaria, AIDS, tetanus and leptospirosis. In these formulations, either an antigen can be fused to flagellin (on its termini or instead of its D3 domain) or the two can be co-administered [207]. The booster effect of flagellins relies on its ability to stimulate TLR5, promoting Th1 and Th2 responses and production of IgM, IgG1, and IgG2c [223,224,225]. Potential obstacles for using flagellin as an adjuvant come from its immunogenicity, and flagellin-specific antibodies could neutralize its adjuvant effects. This could be overcome by removing the variable region. It was shown that *Salmonella* FliC in which D3 and parts of D2 were deleted (Δ174–400) has significantly lower antigenicity while still being able to activate TLR5 [226]. Apart from monomeric flagellin, the entire flagellum could also be used to generate immunity. In the case of *P. aeruginosa*, antibodies against the whole flagella were more effective in preventing infection in mice [227].

Until now, only flagellin-based influenza vaccines, composed of recombinant fusion proteins, have advanced to phase III clinical trials, but none of the flagellin-adjuvanted vaccines has yet received FDA approval for clinical use. Despite potent adjuvant effects induced at low doses of flagellin prior to induction of maximal innate immunity, four of these flagellin-based influenza vaccines (VAX125, VAX102, VAX128, VAX2012Q) induced systemic adverse reactions in clinical studies [228]. Considering these difficulties, Zhao et al. recently reported a promising approach to improving both immunogenicity and safety of flagellin-based vaccines [229]. They designed a construct inserting the full-length flagellin FljB from *S.* *typhimurium* into hepatitis B core protein (HBc) for a high-density display of flagellin onto the surface of virus-like particles (VLPs). Using this strategy they demonstrated that flagellin on the VLP surface significantly reduced its ability to activate TLR5 or induce systemic IL-6 release compared to flagellin alone, which may be due to the D0/D1 domains of flagellin being embedded in the interior of VLPs [229]. The use of active immunomodulation through TLR5 could trigger the release of interferon and inflammatory cytokines, which may aid in minimizing viral replication and serve as a new therapeutic strategy for vaccine or adjuvant development to fight against SARS-CoV-2, as was recently proposed by Chakraborty et al. [230]. More research is needed to understand how TLR5 could act as a modulator of the immune system in order to induce virus-related specific antibodies and to control the infection. Moreover, formulating engineered flagellins with reduced immunogenicity and targeting specific tissues, cells, or signaling pathways will be the next step of innovation in immunologic adjuvants.

### 6.6. Additional Applications of Flagellin

Considering the great variability in the outer domains of flagellin in a single strain alone, as well as the propensity of flagellin for polymerization, it is possible to design filaments that could exhibit a variety of functions. From the late 1980s, numerous studies have confirmed that *Salmonella* and *E. coli* filaments could be used as platforms for peptide display [231]. Moreover, the variable D3 domain in *Salmonella* FliC was successfully replaced by various small proteins such as thioredoxin [232], xylanase A [233], and GFP [234] without affecting its ability to polymerize in vitro or losing the activity of the inserted protein domains. Flagellin monomers and flagellin–protein fusions readily adsorb to hydrophobic surfaces with their D3 domains oriented towards the liquid phase [235], a feature that can be utilized in different ways. For example, Jankovics et al. [236] designed a biosensor by immobilizing a flagellin fusion in which D3 was replaced by the nickel-binding domain of the transcription factor NikR, and successfully used it in binding kinetics studies. Different variants of flagellin can be used to create surface coatings that have either anti-adhesive (in case of the wild type flagellin) or highly adhesive (D3 replaced with a RGD motif) properties [237]. Flagellar filaments can also be used as biotemplates for synthesizing nanomaterials. Such structures can be produced by adding a 12-cysteine tag on the flagellin D3 [238,239], replacing D3 by iron-binding motifs [240], or introducing loops rich with charged residues into the flagellin-thioredoxin fusions [241]. It has been demonstrated that polycations induced assembly of flagellin monomers into nanofilaments, forming thick and smooth composite films through a novel polyelectrolyte multilayer buildup mechanism [242]. These films could find diverse applications in nanotechnology and in biomedical sciences considering the recent discovery of naturally occurring enzymatically active D3 domains of flagellin subunits exposed to the surface of filaments [243].

## 7. Future Perspectives

The bacterial flagellar system is one of the most complex and dynamic proteinaceous structures found in nature. While our knowledge of the filament and its cap has increased considerably in the past few decades, many open questions remain, and we still have only static snapshots that are insufficient to fully understand the process of filament growth. One of the most important questions is how the cap and the filament interact during filament elongation. High flexibility of the FliD leg domains, as well as relatively weak association with the tip, are the main obstacle in obtaining a high-resolution structure that would uncover the atomic details of the FliD-FliC interaction. Since a rigid model of the cap seems improbable, it is necessary to capture many different conformational states of FliD in its native environment.

Another aspect that evades our understanding is the transport of the flagellin monomers through the filament and its folding at the tip. One of the approaches for studying the folded state of flagellin could be fluorescent labeling, which is very challenging in this case because it requires targeted intracellular labeling of the continuously synthesized protein.

One of the most intriguing questions is, however, how to use all this knowledge to solve some of the problems we are facing today. The fact that straight filaments render cells immotile could be exploited in designing small molecules that could lock filaments into the left- or right-handed conformation. These molecules could find application as antimicrobials in combination with existing therapies to prevent the spread of bacteria.

Reprograming bacterial cells for drug delivery using the flagellar system could be even more promising. The use of bacteria in cancer therapy has already been considered and tested in animal models [244]. It is possible to imagine that, after detecting the target location through the bacterial sensory system, bacteria start expressing the protein therapeutic of interest fused to the highly efficient export signal of flagellin, leading to its secretion into the environment.

These potential applications are still far from reality and further research is needed. Nevertheless, we are entering a new phase of flagellar research in which we will actively change parts of the flagellar system or even completely repurpose it in order to address the problems and needs of today and tomorrow.

## Figures and Tables

**Table 1:** Flagellins and FliD and their regulators.

Protein	Function	Sigma Factor	Class in Flagellar Regulatory Hierarchy		Transcriptional Expression Regulators	Post-Transcriptional Regulators	Specific Secretion Chaperone
			3-Tiered	4-Tiered			
Flagellin (FliC, FlaA)	Structural component of the filament	Sigma28 Sigma54 Sigma43	Class III gene	Class IV gene	CodY Environmental factors (nutrients, c-di-GMP, ppGpp, BCAA, temperature…)	Self-regulating CsrA-FliW	FliS
FliD	Filament cap	Sigma70 Sigma28 Sigma54	Class II and III	Class II	Cognate flagellin	?	FliT
